# Differential Bacteriophage Efficacy in Controlling *Salmonella* in Cattle Hide and Soil Models

**DOI:** 10.3389/fmicb.2021.657524

**Published:** 2021-06-28

**Authors:** Yicheng Xie, Tyler Thompson, Chandler O’Leary, Stephen Crosby, Quang X. Nguyen, Mei Liu, Jason J. Gill

**Affiliations:** ^1^Department of Animal Science, Texas A&M University, College Station, TX, United States; ^2^Center for Phage Technology, Texas A&M University, College Station, TX, United States; ^3^Department of Biochemistry and Biophysics, Texas A&M University, College Station, TX, United States

**Keywords:** bacteriophage, *Salmonella*, food safety, antimicrobials, beef cattle

## Abstract

Asymptomatic *Salmonella* carriage in beef cattle is a food safety concern and the beef feedlot environment and cattle hides are reservoirs of this pathogen. Bacteriophages present an attractive non-antibiotic strategy for control of *Salmonella* in beef. In this study, four diverse and genetically unrelated *Salmonella* phages, Sergei, Season12, Sw2, and Munch, were characterized and tested alone and in combination for their ability to control *Salmonella* in cattle hide and soil systems, which are relevant models for *Salmonella* control in beef production. Phage Sergei is a member of the genus *Sashavirus*, phage Season12 was identified as a member of the *Chivirus* genus, Sw2 was identified as a member of the T5-like *Epseptimavirus* genus, and Munch was found to be a novel “jumbo” myovirus. Observed pathogen reductions in the model systems ranged from 0.50 to 1.75 log_10_ CFU/cm^2^ in hides and from 0.53 to 1.38 log_10_ CFU/g in soil, with phages Sergei and Sw2 producing greater reductions (∼1 log_10_ CFU/cm^2^ or CFU/g) than Season12 and Munch. These findings are in accordance with previous observations of phage virulence, suggesting the simple ability of a phage to form plaques on a bacterial strain is not a strong indicator of antimicrobial activity, but performance in liquid culture assays provides a better predictor. The antimicrobial efficacies of phage treatments were found to be phage-specific across model systems, implying that a phage capable of achieving bacterial reduction in one model is more likely to perform well in another. Phage combinations did not produce significantly greater efficacy than single phages even after 24 h in the soil model, and phage-insensitive colonies were not isolated from treated samples, suggesting that the emergence of phage resistance was not a major factor limiting efficacy in this system.

## Introduction

In the United States, foodborne illnesses caused by *Salmonella* are estimated to number more than 1.2 million each year, with more than 23,000 hospitalizations and 450 deaths (Scallan et al., 2011); culture-confirmed *Salmonella* incidence has ranged from 10.9 to 14.8 cases per 100,000 population from the years 2001–2016 (Centers for Disease Control, 2016). *Salmonella* is associated with a wide range of food commodities and about 4% of commercial ground beef in the United States is contaminated with *Salmonella* (Bosilevac et al., 2009), and ground beef contributed to almost a quarter of beef-related *Salmonella* outbreaks from 1973 to 2011 (Laufer et al., 2015). Lymph nodes, which are commonly present in lean trimmings destined for ground beef production, can harbor *Salmonella* without the animal displaying symptoms of illness (Gragg et al., 2013), indicating asymptomatic carriage of *Salmonella* in the lymph nodes of cattle contributes to contamination of ground beef (Bosilevac et al., 2009). *Salmonella* serovars Anatum, Montevideo, and Muenchen are among those most consistently recovered from the lymph nodes of cattle (Gragg et al., 2013; Belk et al., 2018; Nickelson et al., 2019). The lymph nodes of cattle may be colonized by *Salmonella* via oral intake of contaminated water or feeds, or via insect bites or skin abrasions (Pullinger et al., 2007; Edrington et al., 2013; Brown et al., 2015; Olafson et al., 2016). Previous work has shown that the feedlot environment is a reservoir of *Salmonella* that could subsequently contribute to the colonization of lymph nodes of cattle (Gragg et al., 2013; Xie et al., 2016; Belk et al., 2018).

Bacteriophages (phage) are viruses that infect bacteria, and are the most abundant biological entities on earth, estimated to number some 10^31^ to 10^32^ virions (Brussow, 2005; Barr et al., 2013). Phages are ubiquitous in natural environment as well as in plants and animals as a part of their normal flora. Feeding environments of both beef and dairy cattle were previously identified as reservoirs of *Salmonella* phages (Switt et al., 2013; Xie et al., 2016; Duenas et al., 2017). The increasing spread of bacterial resistance to antibiotics has become a worldwide threat, resulting in a renewal of interest in exploring bacteriophage as a potential alternative to control pathogenic bacteria in Western countries (Sulakvelidze, 2011).

The U.S. Department of Agriculture (USDA), Food and Drug Administration (FDA) and Environmental Protection Agency (EPA) have approved phage-based commercial products that are now available as antimicrobial interventions in pre-harvest and post-harvest food production (Goodridge and Bisha, 2011). Previous studies have shown that *Salmonella* phages are able to reduce bacterial loads in poultry (Atterbury et al., 2007; Hungaro et al., 2013; Grant et al., 2017), swine (Harris, 2000), seafood (Galarce et al., 2014), beef (Bigwood et al., 2008), produce (Bai et al., 2019), and multiple other foods (Islam et al., 2020) which suggests a role for phages as an antimicrobial intervention against *Salmonella* from “farm to fork” in food production (García et al., 2010; Sillankorva et al., 2012).

The generic term “phages” refers to an extremely diverse set of organisms; it is not unusual for different phages infecting a single strain of bacteria to have no recognizable sequence similarity. When large and diverse collections of phages are available, a question arises as to which phages should be selected for use as antibacterials. Multiple *in vitro* assays exist for evaluating phage host range and potential efficacy, including assays measuring plaque formation (Khan Mirzaei and Nilsson, 2015), clearance of liquid cultures (Xie et al., 2018), or genome copy number (Gayder et al., 2019). In this study we are interested in determining the relationship between performance in liquid culture, plaque formation, and actual efficacy, using cattle hides and feedlot soil as models of “real world” application systems. Four characterized and genomically unrelated phages capable of infecting the same *S.* Anatum host were selected for use.

Phages are capable of targeting bacterial hosts with high specificity by recognizing unique bacterial surface structures. This specificity reduces collateral damage to other resident microbiota but also allows the target bacteria to become phage resistant by mutational loss of the receptor, which prevents bacterial adsorption (Gill and Hyman, 2010). Phages of *Salmonella* are able to utilize lipopolysaccharides (LPS), outer membrane proteins (OMPs) and flagella as receptors (Lindberg, 1973). It has been suggested that using a cocktail composed of two or more phages targeting different receptors can delay or inhibit the emergence phage resistance (Gill and Hyman, 2010; Bull et al., 2014) and this has been demonstrated in some systems (Yoichi et al., 2005; Bai et al., 2019). Therefore, cross-resistance characterization of individual phages is a key step in rationally developing phage cocktails that are capable of overcoming bacterial insensitivity (Gill and Hyman, 2010).

In this study, we characterize four genetically unrelated lytic *Salmonella* phages and examine their patterns of cross-resistance to formulate phage cocktails capable of overcoming phage resistance. We then study their efficacy *in vitro* and in two model systems with relevance for the potential control of *Salmonella* transmission in the beef cattle feedlot, with the intention of improving the microbiological safety of ground beef.

## Materials and Methods

### Bacterial Strains and Culture Conditions

*S.* Anatum strain FC1033C3 was isolated previously in fecal samples from a cattle feedlot located in south Texas (Xie et al., 2016). *S*. Montevideo strain USDA3 and *S.* Newport USDA2 used for phage propagation were obtained from T. Edrington (USDA, College Station, TX, United States). Bacteria were cultured on trypticase soy broth (TSB, Becton-Dickinson) or trypticase soy agar [TSA, TSB plus 1.5% w/v Bacto agar (Becton-Dickinson)] aerobically at 37°C. A nalidixic acid-resistant derivative of *S.* Anatum FC1033C3 was obtained by plating an overnight bacterial culture on TSA supplemented with 25 mg/L nalidixic acid and selecting for surviving colonies. Bacterial inocula used in the hide and soil models was prepared in peptone water (0.1% peptone (w/v), Becton, Dickinson and Co., Franklin Lakes, NJ, United States). Xylose lysine deoxycholate agar (XLD, Becton-Dickinson) supplemented with 25 mg/L nalidixic acid and 0.1% (w/v) cycloheximide was used to recover *Salmonella* in the hide and soil models.

### Bacteriophage Strains and Culture Conditions

The initial isolation of phage Sergei, Season12, Munch and Sw2 was described in a previous study (Xie et al., 2016). Sergei and Sw2 were isolated on *S*. Anatum FC1033C3, Season12 was isolated on *S*. Newport USDA2 and Munch was isolated on *S*. Montevideo USDA3. Phage stocks were enumerated by the soft agar overlay method as described previously (Xie et al., 2018). High-titer phage stocks were prepared by propagating phage on their respective host strains (Xie et al., 2018) by the confluent plate lysate method (Adams, 1959). For use in the hide and soil models, phage lysates were centrifuged at 8,000 × *g*, 4°C, for 16–18 h and phage pellets gently were resuspended in phage buffer (100 mM NaCl, 25 mM Tris–HCl pH 7.4, 8 mM MgSO_4_, 0.01% w/v gelatin) and stored at 4°C. Phage stocks were adjusted in phage buffer to achieve concentrations of 10^8^ and 10^9^ PFU/mL before use in the hide and soil models.

### Transmission Electron Microscopy Imaging

Transmission electron microscopy of phages was performed by staining virions with 2% uranyl acetate and imaging in a JEOL 1200 EX transmission microscope operating at an acceleration voltage of 100 kV as previously described (Valentine et al., 1968; Gill et al., 2011). Head dimensions and tail length were measured using ImageJ (Schneider et al., 2012; Piya et al., 2017) and standardized against images of a carbon grating replica of known dimensions (Ted Pella, cat# 607). Virion head height was measured from vertex to vertex from the top of the tail to the top of the head, and head width was measured face to face perpendicular to the axis of the tail.

### Genomic DNA Extraction, Sequencing and Bioinformatic Analysis

Phage genomic DNA was prepared by using a modified Wizard^®^ DNA Clean-Up System (Promega, Madison, WI, United States) (Carmody et al., 2010; Gill et al., 2011) and stored at 4°C. Phage genomic DNA was sequenced as paired-end 250 bp reads using the Illumina MiSeq platform. Read quality control, read trimming, and read assembly was achieved by FastQC (bioinformatics.babraham.ac.uk), FastX Toolkit (hannonlab.cshl.edu), and SPAdes 3.5.0 (Bankevich et al., 2012) respectively. PCR and Sanger sequencing of the products was used for genome closure. Glimmer3 (Delcher et al., 1999) and MetaGeneAnnotator (Noguchi et al., 2008) were used to predict protein coding genes with manual correction, and tRNA genes were predicted via ARAGORN (Laslett and Canback, 2004). Putative protein functions were assigned based on sequence homology detected by BLASTp (Camacho et al., 2009) and conserved domains detected by InterProScan 5 (Jones et al., 2014) and HHpred (Zimmermann et al., 2018). Bioinformatic analyses were performed via CPT Galaxy (Cock et al., 2013) and WebApollo (Lee et al., 2013) interfaces (cpt.tamu.edu).

### Characterization of Cross Resistance of Phages

Phage-resistant mutants of *S.* Anatum FC1033C3 were selected by co-culturing the bacterium with a large excess (∼10^8^ PFU, MOI ∼10) of each of the four phages in soft agar overlays as described above and isolating surviving bacterial colonies. Phage-insensitive mutants were subcultured three times. Phage insensitivity was determined by measuring phage efficiency of plating (EOP) on each phage-resistant strain and the phage-sensitive parental strain to determine if resistance to one of the test phages conferred resistance to other phages in the collection (Xie et al., 2018). Phage activity against the parental strain FC1033C3 was measured alone and in combination in a microplate-format assay. A mid-log culture (OD_550__nm_ ∼0.5) was diluted 1,000-fold in TSB and aliquoted to a sterile 96-well microtiter plate at 180 μL per well. Wells were then inoculated with 20 μL of single phages or phage mixtures to final concentrations of 10^8^ and 10^6^ PFU/mL. The plates were incubated at 37°C with double orbital shaking in a Tecan Spark 10M plate reader (Tecan Group Ltd., Männedorf, Switzerland) and growth was monitored by measuring OD_550__nm_ at 30-min intervals for 12 h. Growth curves were achieved by plotting OD after baseline adjustment against time. Three biological replicates were performed for each condition.

### Cattle Hide Model

An overnight culture of the nalidixic acid-resistant *S.* Anatum strain FC1033C3 was centrifuged, washed three times with peptone water and inoculated into a sterile gelatin-based slurry at a final concentration of ∼ 6.8 log_10_ CFU/mL to mimic the adherent properties of soil and fecal contamination (Villarreal-Silva et al., 2016). Cattle hide pieces were obtained from Texas A&M Rosenthal Meat Science and Technology Center during harvest with a circular punch to achieve hide swatches with an average surface size of 70 cm^2^. Five mL of slurry was applied to the freshly collected cattle hide swatches and allowed 30 min of contact time at 37°C, followed by removal of excess material with a plastic cell spreader. Inoculated hide pieces were sprayed with 5 mL of individual phages or phage combinations at concentrations of 10^8^ or 10^9^ PFU/mL and held at 37°C for 1 h in a humidified environment. Phages Sergei, Season12, Munch and SW2, representing four distinct phage types, were used; two phage combinations, Sergei + Munch and Sergei + Sw2, were also evaluated, based on the lack of cross-resistance shown by these phages (see below and Table 2). Sham treatments were performed as controls by spraying 5 mL of phage buffer onto inoculated hide pieces. Treated hide pieces were placed in filtered stomacher bags with 100 mL of peptone water and homogenized in a stomacher for 60 s, and 5 mL of homogenized mixtures were centrifuged at 8,000 × *g* for 10 min and pellets were resuspended in 5 mL peptone water, serially diluted and spread on XLD supplemented with 25 mg/L nalidixic acid and 0.1% cycloheximide. Plates were incubated at 37°C for 18 h and colonies enumerated. Each experiment was performed in three biological replicates.

**TABLE 1:** Phage virion dimension and general genome characteristics.

Bacteriophage	Morphology			Genome		
	Head (nm)	Tail (nm)	Morphotype	Length (bp)	Genus	GC Content (%)
Sergei	69 × 63	156 × 11	Sipho	56,051	*Sashavirus*	43.4
Season12	57 × 54	231 × 12	Sipho	57,039	*Chivirus*	56.5
Munch	119 × 99	125 × 23	Myo	350,103	Unclassified	35.6
Sw2	77 × 72	185 × 8	Sipho	114,274	*Epseptimavirus*	40.2

*Virion head dimensions are displayed as head height × head width. Virion tail dimensions are displayed as tail length × tail width.*

**TABLE 2:** Phage efficiencies of plating (EOP) on the parental strain *S.*Anatum FC1033C3 and its four phage-insensitive derivatives.

Bacteriophage	Bacterial Strain				
	*S.* Anatum FC1033C3	Sergei^R^	Season12^R^	Munch^R^	Sw2^R^
Sergei	1.00	<10^–7^	0.98	0.75	0.70
Season12	1.00	1.10	<10^–7^	<10^–7^	1.15
Munch	1.00	0.02	0.02	<10^–7^	0.02
Sw2	1.00	1.17	1.42	1.42	<10^–7^

*EOP’s are normalized to the titer of each phage on the parental strain. Mutant strains are insensitive to the phages used to select them and have varying sensitivities to the other three phages tested.*

### Soil Model

The bacterial inoculum was prepared and washed in peptone water as described above. Soil was collected from a cattle feedlot located in College Station, TX and soil compositional analysis was performed at the Soil, Water and Forage Testing Laboratory at Texas A&M University. Soil was determined to be composed of 55% sand, 28% silt, and 17% clay. Silica sand was obtained from a commercial supplier (Standard Sand, Millipore-Sigma). Soil and sand were sterilized by autoclaving three times at 121°C for 30 min, and 10 g aliquots of sterilized materials were placed into plastic 90 mm Petri plates. Soil or sand was inoculated by spraying 3 mL of inoculum, and allowing 30 min of contact time at 37°C. Inoculated samples were sprayed with 3 mL of individual phages or phage combinations as appropriate at concentrations of 10^8^ or 10^9^ PFU/mL and held at 37°C for 1 or 24 h treatment periods in a humidified environment. Sham treatments were performed as controls by spraying 3 mL of phage buffer onto inoculated sand and soil samples in Petri plates. Treated samples were placed in filtered stomacher bags with 100 ml of peptone water and homogenized by hand massage for 60 s, and 5 mL of homogenized mixtures were centrifuged at 8,000 × *g* for 10 min and pellets were resuspended in 5 mL peptone water, serially diluted and spread on XLD agar supplemented with 25 mg/l nalidixic acid and 0.1% cycloheximide. Plates were incubated at 37°C for 18 h and colonies enumerated. Each experiment was performed in three biological replicates.

### Statistical Analysis

Bacterial survival from different phage treatments in the hide or soil models were analyzed for differences between treatments by one-way analysis of variance (ANOVA) at α = 0.05 via JMP v12.1.0 (JMP^®^ Statistical Discovery^TM^ From SAS, Cary, NC, United States). Significantly different bacterial concentrations between treatments were determined by pairwise Student’s *t*-tests (*P* < 0.05).

### Accession Numbers

Phage genomes are deposited in NCBI GenBank under the following accession numbers: KY002061 (Sergei); MK286578 (Season12); MK268344 (Munch); MH631454 (Sw2).

## Results

### Bacteriophage Characterization

**FIGURE 1:**
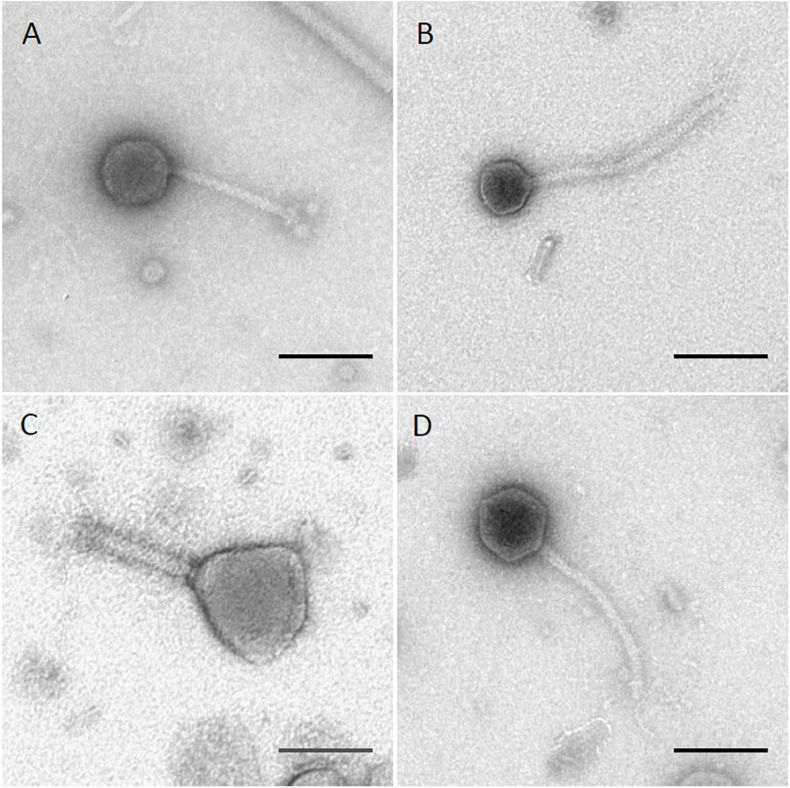
TEM images of four *Salmonella* phages. **(A)** phage Sergei; **(B)** phage Season12; **(C)** phage Munch; **(D)** phage Sw2. Black lines indicate 100 nm for scale.

The four phages used in this study were previously isolated as described (Xie et al., 2016, 2018). Results of transmission electron microscopy are shown in Figure 1, with phages Sergei, Season12, and Sw2 exhibiting typical siphophage morphology with long, non-contractile tails and phage Munch exhibiting myophage morphology with a long, contractile tail and a slightly prolate head. The phage Munch virion is exceptionally large, with a head width of 99 nm. All phage genomes were sequenced to completion on the Illumina platform, and closure was confirmed by PCR. Genome maps of Season12, Munch and Sw2 are shown in Supplementary Figures 1–3, respectively. A genome map of Sergei is shown in Zeng et al. (2019). Phage dimensions and general genome characteristics are summarized in Table 1.

Phage Sergei belongs to a group of closely related phages similar to the *Salmonella* phage 9NA (KJ802832) that was proposed as a new virus genus (Casjens et al., 2014; Zeng et al., 2019); DNA-level similarity to other related phages places Sergei as likely its own species within the *Sashavirus* genus, with 93.5% similarity to *Salmonella* phage Sasha (NC_047786) as determined by progressiveMauve (Darling et al., 2010). Phage Sergei has a genome of 56,051 bp with a GC content of 43.5%. The genome of phage Sergei is presumably terminally redundant and circularly permuted based on its close relationship to phage 9NA; the annotation of this genome and its relatives is described in detail elsewhere (Zeng et al., 2019).

Phage Season12 was found to be 91% identical to *Salmonella* phage Chi (KM458633) at the DNA level determined by progressiveMauve, placing it in the *Chivirus* genus. The genome of Season12 is 59,059 bp in length with a GC content of 56.5%. The genome assembled into a single linear contig at 34.8-fold coverage starting with a 12-bp predicted 5′-overhang cohesive (*cos*) end (5′-GGTGCGCAGAGC-3′) that is conserved in phage Chi and other Chi-like phages (Hendrix et al., 2015; Leavitt et al., 2017). There are 76 protein coding genes predicted in the genome of Season12, in which 50 are of unknown function. Genes associated with DNA replication and transcription, such as DNA primase, DNA polymerase and DNA helicase, and structural proteins such as the portal, major capsid protein and major tail protein share high degree of identity (∼80 – 99%) with Chi. The predicted Season12 tail fiber protein is >99% identical to that found in Chi, with only two amino acid variations. No tRNA genes were detected in Season12. Three tandem repeats (protein identity ranging from 24 to 37%) of a gene encoding a hypothetical protein were identified downstream of the Season12 tail fiber protein. This tandem gene repeat is also found in the genomes of closely related phages such as Chi, iEPS5 (KC677662) and SPN19 (JN871591).

Phage Munch is a so-called “jumbo” phage with a large genome of 350,103 bp and a relatively low GC content of 35.6%. The genome was sequenced to 12.4-fold coverage and was predicted to be terminally redundant and non-permuted by PhageTerm (Garneau et al., 2017); the genome was reopened at the boundary of a predicted 21,296 bp direct terminal repeat identified by this tool. The “jumbo” phage classification is an informal grouping that includes a diverse set of phages with genomes greater than ∼200 kb (Yuan and Gao, 2017). The phage Munch genome has 532 predicted protein coding genes and 22 tRNAs. Of these predicted protein coding sequences, only 118 could be assigned a putative function. Like many jumbo phages, Munch is not closely related to any other known phage; the low DNA sequence identity to other phage genomes (<30% as determined by progressiveMauve) indicates Munch is likely the founding member of an as-yet unclassified myoviral genus. At the protein level, the most closely related phage to Munch is phage 121Q (KM507819), which shares 270 proteins with Munch based on a BLASTp analysis with an *E* value cutoff of 0.001. Other related phages include vB_Eco_slurp01 (LT603033, 268 shared proteins), vB_CsaM_GAP32 (JN882285.1, 265 shared proteins) and PBECO 4 (KC295538.1, 264 shared proteins).

Three regions containing repeated DNA sequence were identified in the Munch genome by Dotmatcher (Supplementary Figure 4; Cock et al., 2013). Protein sequences within these regions were further compared using BLASTp. Genes from the repeat region located in the first 20 kb of the genome did not display detectable similarity in protein sequence, suggesting that if these proteins are the result of gene duplication, this event would have occurred in the distant past. The second repeat region was located completely within the tail fiber protein gene (position 140,830–145,512) (Supplementary Figure 2), however, no obvious repeated protein motif was identified in this gene product. The final repeat region which spans the right-most ∼20 kb of the genome contains 13 tandem repeats of a gene encoding a predicted DNA condensation protein (IPR009091).

The 114,274 bp genome of phage Sw2 was sequenced to 73.5-fold coverage. Sw2 has a GC content of 40.2% and the genome was reopened to a predicted 8,123 bp non-permuted terminal repeat as determined by PhageTerm (Garneau et al., 2017). Phage Sw2 shares 59% DNA sequence identity with the well-studied *Siphoviridae* phage T5 (NC_005859.1), and contains 197 predicted protein coding genes, of which 82 could be assigned a function. There are 29 tRNA sequences annotated in the genome of Sw2. The current ICTV taxonomy of phages has classified Sw2 as its own species in the genus *Epseptimavirus*.

### Characterization of Phage-Resistant Mutants

Mutants of *S*. Anatum FC1033C3 insensitive to phages Sergei, Season12, Munch, and Sw2 were obtained by culturing the bacterium with an excess of each phage. The efficiency of plating (EOP) of each phage was determined on each insensitive mutant standardized to the phage titer obtained by plating on the parental strain FC1033C3 (Table 2). Phage Sergei and Season12 were able to infect each other’s phage-insensitive mutants with EOP’s close to 1, showing that Sergei and Season12 are genetically independent for phage resistance. In contrast, phage Munch was able infect the Season12-insensitive mutant at a roughly 100-fold reduced EOP but Season12 was completely unable infect the Munch-insensitive mutant, showing a partially dependent phenotype for phage insensitivity. Phage Munch exhibited this 100-fold reduction in EOP on Sergei, Season12 and Sw2-insensitive mutants, suggesting that Munch infection is easily perturbed by the development of insensitivity to multiple other phages.

### Efficacy Testing on Single and Mixed Phages Against *S.* Anatum FC1033C3 in a Microtiter Plate Liquid Assay

Because phages select for phage-insensitive mutants at relatively high frequencies, it was hypothesized that using a combination of phages that are genetically independent for phage insensitivity can improve antimicrobial efficacy (Gill and Hyman, 2010). Based on the results shown in Table 3, a pair of two-phage cocktails were formulated that contained phages that were either genetically independent for insensitivity (Sergei and Munch) or partially dependent (Munch and Season12). The performance of these phage cocktails was evaluated in a microtiter plate assay and compared to the performance of the individual phages.

**TABLE 3:** Comparison of two methods for measuring phage virulence and the observed phage efficacy in reducing *S.* Anatum loads in two model systems.

Phage isolate	Phage virulence scores		Reductions in model systems with phage concentration at 10^9^ PFU/ml		
	Spot assay (EOP)^1^	Microtiter assay (10^8^ PFU/ml)^2^	Cattle hide (Log_10_ CFU/cm^2^)	Soil (Log_10_ CFU/g)	
				1 h	24 h
Sergei	0.5–1	55	1.60^*^	1.38^*^	0.45^*^
Season12	0.5–1	10	0.50	0.22	0.33
Munch	0.5–1	27	0.83^*^	0.53^*^	0.33
Sw2	0.05–0.5	86	1.75^*^	0.77^*^	0.88^*^

*Asterisks by log_10_ reductions in model systems indicate statistically significant reductions compared to the control (*P* < 0.05). Host range scores from Spot Assays and Microtiter Assays are taken from Xie et al. (2018). Note that estimation of phage virulence based on plaque formation (Spot Assay) is a poor predictor of phage efficacy in the hide and soil model systems; phages with higher virulence in liquid culture (Microtiter Assay) tend to perform better in the model systems.*

*^1^An EOP of ∼0.5 – 1 corresponds to a score of 4, and an EOP of ∼0.05 – 0.5 corresponds to a score of 3, as described in Xie et al. (2018).*

*^2^The microtiter plate assay was conducted by monitoring bacterial growth by optical density in the presence of phage in a 96-well microtiter plate. The score is equal to the area between bacterial growth observed in the positive (no phage) control and the phage-treated culture. A score of zero would indicate complete insensitivity to the phage and a score of 100 would indicate complete elimination of bacterial growth. Method and results are described in Xie et al. (2018).*

**FIGURE 2:**
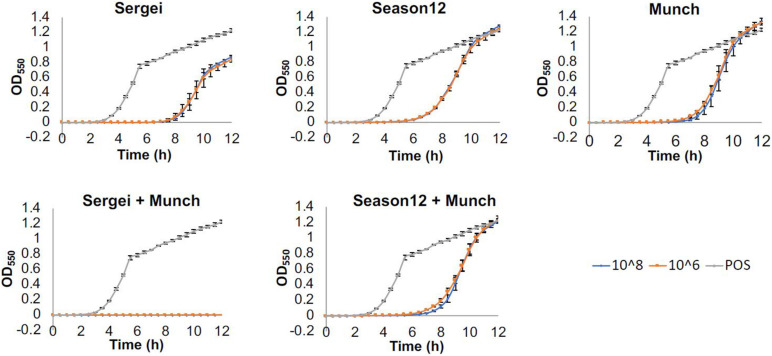
Efficacies of phages alone and in combination against *S.* Anatum FC1033C3 in a microtiter-plate based assay. Wells of 96-well microtiter plates were inoculated with bacterial culture (∼10^5^ CFU/mL) and challenged with single phages or phage mixtures at concentrations of 10^8^ or 10^6^ PFU/mL, and growth was monitored by measuring OD_550nm_ at 30-min intervals for 12 h. Growth curves were achieved by plotting OD after baseline adjustment against time. Three biological replicates were performed for each assay. Blue, orange and gray curves represent growth profiles of phage treatments at 10^8^ PFU/mL, phage treatments at 10^9^ PFU/mL and positive (no phage) control.

Results obtained from this experiment are shown in Figure 2. When testing phages Sergei, Munch or Season12 alone against the wild type *Salmonella* strain, regrowth of bacterial culture was observed starting at 7, 6, and 5 h, respectively. This observation of bacterial regrowth is consistent with the rise of phage-insensitive mutants in the culture. By using a combination of two phages with genetically independent resistance (Sergei and Munch), no regrowth was observed during the 12-h experiment, demonstrating a significant improvement of antimicrobial efficacy against the test *Salmonella* strain. In contrast, by using a combination of two phages with partially dependent resistance (Season12 and Munch), no improvement of antimicrobial efficacy over the individual phage was observed.

### Ability of Phages to Reduce *Salmonella* Populations in Model Systems

**FIGURE 3:**
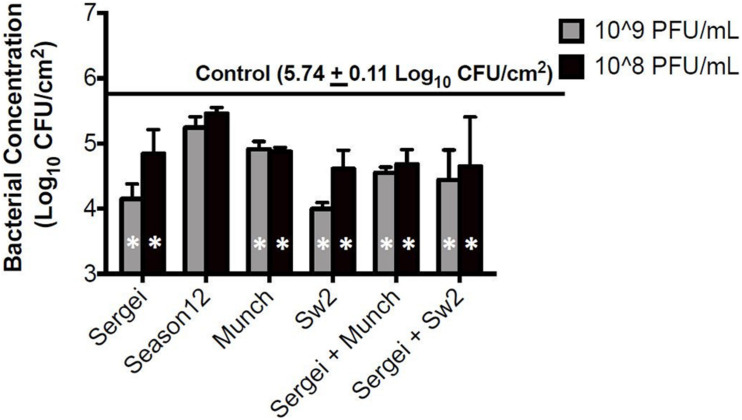
Bacterial survival after phage treatments in the cattle hide model. Bars indicate bacterial survival after 1 h phage treatment. Bar shading indicates two phage treatment concentrations at 10^8^ and 10^9^ PFU/mL, and the solid line parallel to the *X*-axis indicates the bacterial load in the positive (no phage) control. Error bars indicate standard deviation across three biological replicates. White stars in the bars represent significant difference in the treatment from the positive control. The detection limit of this assay is 1.15 log_10_ CFU/cm^2^.

Treatments with single phages and mixtures of phages with genetically independent resistance were tested against *S.* Anatum FC1033C3 in model systems of *Salmonella*-contaminated cattle hides and soil, two common reservoirs for *Salmonella* in the beef cattle feedlot (Gragg et al., 2013; Xie et al., 2016). In the hide model, all phage treatments except phage Season12 were able to significantly reduce *Salmonella* populations on cattle hides compared to the positive control (Figure 3) at 5.74 log_10_ CFU/cm^2^. A reduction of 1.75 log_10_ CFU/cm^2^ was obtained by phage Sw2 alone at 10^9^ PFU/mL, which was the highest bacterial reduction among all treatments performed. Limited dosage effects were observed across phage treatments, with statistically significant differences only observed between the two phage treatment concentrations in phages Sergei and Sw2. No significant differences were observed between the phage mixtures (Sergei + Munch, Sergei + Sw2) and phage Sergei alone, indicating the phage mixtures provided no added efficacy over using a single phage.

**FIGURE 4:**
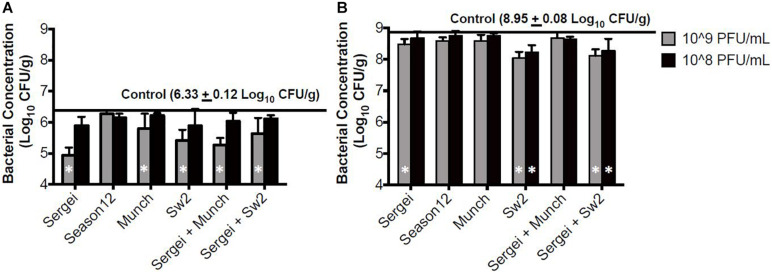
Bacterial survival after phage treatments in the sterile soil model. **(A,B)** indicate bacterial survival following phage treatments of 1 and 24 h, respectively. Bar shading indicates two phage treatment concentrations at 10^8^ and 10^9^ PFU/mL, and the solid line parallel to the *X*-axis indicates the bacterial load in the positive (no phage) control. Error bars indicate standard deviation across three biological replicates. White stars in the bars represent significant difference in the treatment from the positive control. The detection limit of this assay is 2 log_10_ CFU/g.

In the sterile soil model sampled 1 hr post-treatment, statistically significant reductions were observed only with phage concentrations at 10^9^ CFU/mL for phages Munch, Sergei and Sw2 alone, and with phage mixtures Sergei + Sw2 and Sergei + Munch (Figure 4A). Phage Sergei was able to reduce the bacterial load from 6.33 log_10_ CFU/g (control) to 4.95 CFU/g, and Sergei alone and its combination with Munch showed a statistically significant dosage effect (*P* < 0.05). At the 24 h sampling time, the bacterial load in the soil had grown to 8.95 log_10_ CFU/g in the control treatment, and phage Sw2 and its combination with Sergei was able to significantly reduce the bacterial population in soil compared to the control treatment at either treatment concentration. Sergei alone significantly reduced bacterial load only when applied at 10^9^ PFU/mL. Phage treatment with Sw2 at a concentration of 10^9^ PFU/mL resulted in the greatest reduction in this model of log_10_ 0.82 CFU/g. To examine the role of phage resistance in this model, 60 *S.* Anatum colonies were recovered from soils following treatment with phage Sergei and Sergei + Munch in one of the experimental replicates. Of the 10 colonies recovered from each phage treatment after 1 h, and the 20 colonies recovered from each treatment after 24 h, none were resistant to either phage Sergei or Munch, indicating that the arisal of phage-resistant bacterial mutants was not a significant issue in this system (Supplementary Table 1).

**FIGURE 5:**
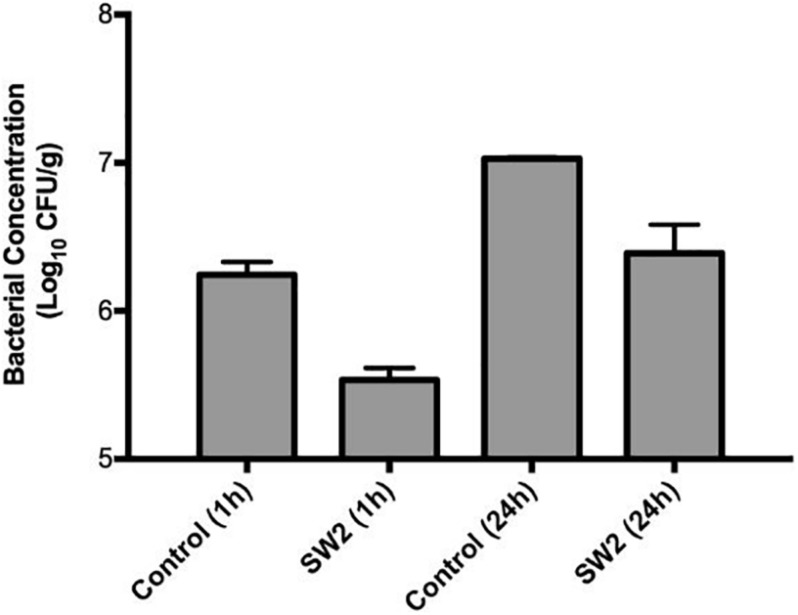
Bacterial survival in a sterile sand model after treatment with sterile buffer (control) or phage Sw2 at 10^9^ PFU/mL. Bacterial loads were measured 1 and 24 h after treatment. The detection limit of this assay is 2 log_10_ CFU/g.

Comparing the bacterial survival between these two models, treatments with phages appeared to demonstrate better efficacies in the hide model than the soil model. Phages are known to interact with charged particles in soils which may interfere with their ability to efficiently diffuse and locate their hosts (Bitton, 1975). To further examine this system, phage Sw2 was applied to a sterile quartz sand model inoculated under the same conditions as the soil model. The sand model was intended to mimic the physical structure of soil while providing a more uniform substrate (Redman et al., 1999). Results from the sand model are displayed in Figure 5. Sw2 was able to significantly reduce the *Salmonella* population by 0.8 log_10_ CFU/g at the 1 hr sample time and continued to suppress bacterial growth by 0.64 log_10_ CFU/g at 24 h; both of these reductions are statistically significant compared to the control treatments. The sand model supported reduced bacterial growth compared to the soil model, reaching only 7.0 log_10_ CFU/g after 24 h compared to the 8.95 log_10_ CFU/g reached in the soil model. However, the log_10_ reductions in bacterial loads produced by phage in the sand model were not markedly better than those observed in the soil model (Figure 5).

## Discussion

### Characterization of Phages

With the exception of phage Munch, phage genomes showed high levels of similarity to other well-known phage types. Conserved structural proteins found in phages Sergei, Season12, and Sw2 provided useful information to understand the dynamics of phage infection. In particular, the characterization of phage tail fiber proteins is useful for prediction of phage receptors and interpretation of phage cross-resistance patterns.

Sergei is related to phage 9NA, and is a member of the *Sashavirus* genus. The structural proteins of Sergei, including the portal protein, major capsid proteins and major tail proteins share high (∼90%) identity within this phage group. Phage Sergei encodes two predicted receptor-binding proteins, a tail tip protein (gp60, APU92897) with a lambda J-like conserved domain and a tailspike protein (gp61, APU92898). The tail tip protein is conserved in 9NA-like phages including Sasha, Sergei and 9NA (Zeng et al., 2019). The tailspike protein is related to the 9NA tailspike (gp55, YP_009101225) but its sequence is conserved only at the N-terminus, with an unrelated C-terminal domain. The Sergei gp61 C-terminal domain contains a pectate lyase fold domain (IPR011050) and is related to predicted tail fiber proteins found in other *S.* Anatum prophages, suggesting Sergei also uses the bacterial LPS as its receptor (Zeng et al., 2019).

Phage Season12 is a member of the *Chivirus* genus, with strong DNA sequence similarity to phage Chi itself. Bacteriophage Chi was first isolated in 1936 and is known for its use of bacterial flagella to infect *Salmonella* spp. (Meynell, 1961). The adsorption mechanism of Chi-like phages has been studied in related phages iEPS5 (Choi et al., 2013) and YSD1 (Dunstan et al., 2019). Choi et al. (2013) indicated that phage iEPS5 were only able to infect the bacterial host when the flagellum is rotating counterclockwise, suggesting the physical movement of flagellum generates power that drives phages toward to the bacterial surface. The overall similarity of phage Season12 to Chi and their nearly identical (96% identity) major tail fiber proteins (gp31 in both phages) strongly suggests the use of the flagellum as the major receptor of Season12.

Phage Munch has an unusually large genome of >350 kb, and a head diameter of ∼100 nm, making it the largest *Salmonella* phage isolated to date. The term “jumbo phage” is generally given to phages with genome sizes larger than 200 kb (Yuan and Gao, 2017), and like many such phages, a large proportion of its genes (78%) could not be annotated with a known function. The majority of jumbo phages have been isolated against GC-rich Gram-negative hosts, but these phages tend to display an AT-rich genome (Mesyanzhinov et al., 2002; Yuan and Gao, 2017); phage Munch is no exception, with an overall GC content of 35.6%. Structural proteins annotated in Munch, such as major capsid proteins, portal proteins and baseplate proteins, are predominantly related to structural proteins found in coliphage T4. Other genes associated with nucleotide metabolism and replication were also annotated, however, no virus-encoded RNA polymerase (RNAP) was identified. Based on protein sequence similarity, phage Munch is most closely related to other jumbo phages such as 121Q (KM507819.1), GAP32 (JN882285.1), and PBECO 4 (KC295538.1). These phages were noted to reside in a single cluster in a recent comparison of 52 jumbo phage genomes (Yuan and Gao, 2017), and these phages appear to be only distantly related to other more well-studied jumbo phages such as phiKZ or KVP40. Phage Munch also has almost no detectable relationship with the 240 kb *Salmonella* jumbo phage SPN3US (JN641803), with only a single SPN3US protein (gp224) having detectable similarity to a Munch protein by BLASTp. A region of tandemly repeated genes was identified in the rightmost ∼20 kb of the Munch genome, in which 13 repeats of a putative DNA condensation protein, separated by three unrelated hypothetical proteins, were identified. This repeat region was not found in any related jumbo phages such as phages 121Q, vB_Eco_slurp01 (LT603033.1), GAP32, PBECO 4, and vB_KleM-RaK2 (JQ513383.1). Tandem gene duplications have been observed in other jumbo phages such as 121Q and G (Hua et al., 2017). Hua et al. (2017) suggested that jumbo phages may expand their genomes though tandem duplications after head size expansion due to a sudden lack of selective pressure to constrain their genome size. The two putative tail fiber proteins in phage Munch (gp252 and gp287) show low levels of homology to those found in other jumbo phages, and the bacterial receptors recognized by these phages are not known.

Sw2 is a member of the T5-like *Epseptimavirus* genus. The Sw2 genome encodes 29 tRNAs, compared to the 16 identified in T5 (Wang et al., 2005). Like other T5-like phages, the genome of Sw2 can be divided into pre-early genes, early and late genes (Wang et al., 2005). Proteins encoded in pre-early genes are associated with host shutdown, including 5′-deoxyribonucleotidase, A1, and A2 (Wang et al., 2005). The early gene cluster functions related to DNA metabolism, replication, regulation, and lysis, followed by late gene region consists of virion structure (Wang et al., 2005). Sw2 encodes an L-shaped tail fiber (gp180), a second putative tail fiber (gp179) and a receptor-binding protein (gp200). In T5, the L-shaped tail fiber (Ltf) recognizes the host O-antigen (Heller and Braun, 1982) and the receptor-binding protein (Oad, also called Pb5) recognizes the outer membrane protein FhuA (Braun et al., 1994). The gp179-gp180 dual tail fiber module in Sw2 is similar to that observed in the T5-like phages DT57C and DT571/2, suggesting this phage may also possess a complex, possibly branched tail fiber structure as reported in these phages (Golomidova et al., 2016). This may explain the relatively broad host range of this phage, which is capable of infecting diverse *Salmonella* serotypes including Anatum, Dublin, and Heidelberg (Xie et al., 2018). The Sw2 receptor-binding protein gp200 is related to its T5 counterpart (*E* = 10^–29^) at the N and C termini, but there is significant divergence between the two sequences (∼27% overall identity). Sw2 gp200 is more closely related to the receptor-binding protein of the T5-like phage BF23 (Hrs, AAZ03642), with 73% overall identity. Phage BF23 utilizes the *E. coli* outer membrane protein BtuB as its receptor (Mondigler et al., 2006), which suggests that phage Sw2 gp200 also recognizes this receptor in its *S*. Anatum host. The presence of these tail proteins in Sw2 suggests that this phage uses a two-stage strategy of reversible followed by irreversible binding similar to that used by phage T5.

Phage binding to the host receptor is the initial step of infection, and bacteria spontaneously develop resistance to phage mainly via loss of receptors (Labrie et al., 2010). Therefore, in phage therapeutic applications, it has been proposed that phage cocktails should be used that recognize independent receptors, to prevent the emergence of phage resistance and improve efficacy (Gill and Hyman, 2010). Based on the phage cross-resistance patterns observed in Table 2, phages Sergei, Season12 and Sw2 recognize genetically independent receptors as they were still able to infect the mutants resistant to the other phages, and phage mixtures that recognize independent receptors showed greater efficacy in liquid culture (Figure 2). The cross-resistance patterns of these phages are generally consistent with phage receptor usage as predicted by genomic analysis, as the recognition of LPS by phage Sergei, for example, is not expected to be affected by loss of the flagella (used by Season12) or BtuB (used by Sw2). An exception to this pattern is seen in the case of phage Munch; this phage shows a ∼100-fold reduced EOP on all phage-resistant mutants, and phage Season12 is also not able to infect the Munch-resistant mutant host (Table 3). This latter effect may reflect a significant cell wall defect associated with resistance to phage Munch that interferes with flagella function, or perhaps loss of a secondary receptor at the cell surface required by Season12 for infection.

### Antimicrobial Activity of Phages in Model Systems

The ability of a phage to rapidly negatively impact a bacterial population, often referred to as “virulence,” is an integrated result of its ability to adsorb to new hosts and release progeny phage (Payne and Jansen, 2001; Gallet et al., 2009; Lindberg et al., 2014). The host ranges and *in vitro* virulence of phages Sergei, Season12, Munch and Sw2 were determined in a previous study using spot assays on soft agar overlays and a microtiter plate-based assay of phage virulence (Xie et al., 2018). While these four phages displayed similar abilities to produce plaques in spot titer assays on soft agar overlays, phages Sergei and Sw2 displayed greater virulence against *S.* Anatum FC1033C3 in liquid culture (Xie et al., 2018). To better demonstrate the correlation between *in vitro* phage virulence and antimicrobial efficacy in model systems, the results obtained for these phages in previous and current studies are summarized in Table 3. Phages Sergei and Sw2 were most effective in controlling bacterial growth in liquid culture, and also achieved the highest bacterial reductions in both the hide and soil model systems, implying that measurement of phage virulence in liquid culture can be a predictor of phage efficacies in other, more complex systems. Phages Season12 and Munch were capable of efficiently making plaques on agar overlays but did not exhibit strong antibacterial activity in either the microtiter plate virulence assay or the two model systems of *S.* Anatum colonization (Table 3). The tendency of spot assays to overestimate phage virulence was also observed by Henry et al. (2013) in a mouse model of *Pseudomonas aeruginosa* infection. A similar trend was also noted by Lindberg et al. (2014), where phage growth rate in liquid culture was a strong predictor of phage *in vivo* efficacy in a *P. aeruginosa* insect infection model. This contrasts with observations by Bull et al. (2012), where two phages with similar *in vitro* virulence displayed markedly different efficacies in a rodent model. This phenomenon was attributed to the ability of one of the phages to produce an additional therapeutic protein, a capsular depolymerase, which attenuates bacterial virulence *in vivo* (Mushtaq et al., 2004; Lin et al., 2017). Taken together, these findings suggest that, barring the presence of emergent effects that are only observable in the model system, the virulence or efficacy of a phage is largely phage-dependent and model-independent. Phages such as Sergei or Sw2 are better at controlling bacterial populations in simple liquid culture systems (Xie et al., 2018) and this ability extended to other more physically complex systems such as animal hides or soil in this study.

While individual phage efficacy was conserved across systems, the overall magnitude of the effect on bacterial populations varied slightly between systems. The phage treatments displayed overall reduced antimicrobial efficiency in the soil model compared to the hide model (Figures 3, 4 and Table 3). Input MOI’s of phage in the soil and hide systems were ∼10 (10^8^ PFU/ml treatments) or ∼100 (10^9^ PFU/ml treatments), providing enough phage to infect >99.99% of the bacterial cells in these systems, yet only ∼1.5-fold to ∼50-fold reductions in *Salmonella* loads were observed (Table 3), suggesting that phages were inhibited in their ability to successfully encounter their bacterial targets in these systems, and this effect was more pronounced in the soil system than the hide system. This lack of phage-host encounters is supported by the complete absence of phage resistance in 40 bacterial colonies recovered from soil 24 h after treatment with phage Sergei or Sergei + Munch, indicating the persistence of *Salmonella* in the phage-treated soil was not due to the emergence of phage resistance (Supplementary Table 1). Phage can adsorb to charged particles found in natural soils (Bitton, 1975; Burge and Enkiri, 1978), which may affect their ability to freely diffuse and encounter new hosts. The ability of phage Sw2 to reduce bacterial loads was also evaluated in a model system of pure quartz sand, which provided a more uniform matrix than natural soil. As shown in Figures 4A, 5, phage Sw2 showed a similar ability to control *Salmonella* in both the sand and soil models at 1 h post-treatment, indicating that phage efficacy in soil is not highly dependent on the composition of a particular soil matrix and suggesting that it is the high surface area and reduced diffusion that play major roles in determining phage activity. This phenomenon would also explain why treatments with phage combinations did not exhibit synergistic effects compared to single phage treatments, in contrast to Bai et al. (2019), who observed significantly greater reductions of *Salmonella* using combinations of receptor-independent phages in produce models which present a relatively smooth surface and less inhibition of phage diffusion.

In addition to information on phage lifestyle and receptor usage, bioinformatic analysis may also provide additional predictive information of phage efficacy. The Chi-like nature of Season12 suggests that it requires an active bacterial flagellum for infection (Schade and Adler, 1967; Hendrix et al., 2015). Conditions in the models tested in this study are sub-optimal for *Salmonella* growth and survival, potentially resulting in a stress response (Shen and Fang, 2012) which can lead to downregulation of genes responsible for flagellar synthesis and decreased motility (Fan et al., 2016). Flagellar loss or lack of flagellar rotation can negatively impact the infection process of Chi-like phages, which may further reduce the efficacy of this type of phage in model systems. In addition, the presentation of *Salmonella* flagella is subject to phase variation (Bonifield and Hughes, 2003), and it is possible that Season12 cannot use these flagellar antigens as receptors with equal efficiency. These observations are consistent with the poor efficacy observed for phage Season12 in both model systems (Figures 3, 4), and may indicate against using flagellar-adsorbing Chi-like phages in applications where flagellar activity may be impaired.

Phages have re-emerged as an attractive alternative to combat pathogenic bacteria that have become resistant to antibiotics, or that colonize sites where the use of chemical antimicrobials is not appropriate. The capacity of phages to efficiently infect and lyse their targets is essential for successful phage therapy (Gill and Hyman, 2010; Nilsson, 2014; Roach and Debarbrieux, 2017), but the ability to predict which phages provide greatest efficacy is still an evolving field. In this study, genomic and microbiological approaches were leveraged to characterize and apply four phages that infect the same *S.* Anatum strain. Genomic analysis showed the phages were unrelated, and predictions of the phage receptors were consistent with observed patterns of phage cross-resistance. Phages that displayed greater virulence in *in vitro* assays achieved best reductions of *Salmonella* loads (up to 1.75 log_10_) in cattle hide and soil models, supporting the concept that phage efficacy tends to be phage-specific rather than model-specific. Reductions in bacterial loads on the order of 10-fold to 50-fold could provide a significant food safety benefit by reducing cattle hide and lymph node carriage and subsequent downstream contamination of beef products.

## Data Availability Statement

The datasets presented in this study can be found in online repositories. The names of the repository/repositories and accession number(s) can be found below: https://www.ncbi.nlm.nih.gov/nucleotide/KY002061; https://www.ncbi. nlm.nih.gov/nucleotide/MK286578; https://www.ncbi.nlm.nih. gov/nucleotide/MK268344 and https://www.ncbi.nlm.nih.gov/nucleotide/MH631454.

## Author Contributions

YX and JG conceived of the study. YX conducted phage isolation and *in vitro* experiments with the assistance of TT, CO’L, and ML. YX and ML sequenced the phage genomes. SC, QN, ML, and JG annotated the genomes and conducted bioinformatic analyses. YX, ML, and JG assembled the manuscript. All authors contributed to the article and approved the submitted version.

## Conflict of Interest

JG consultant, Merck & Co. (June 2019); Member, Scientific Advisory Board, Deerland Enzymes Inc. YX currently employed at Bio-Rad, Hercules, CA. The remaining authors declare that the research was conducted in the absence of any commercial or financial relationships that could be construed as a potential conflict of interest.
