# Supplementary material for: Differential Bacteriophage Efficacy in Controlling Salmonella in Cattle Hide and Soil Models
Source: Front Microbiol. 2021 Jun 28;12:657524. doi: 10.3389/fmicb.2021.657524 (PMC8273493; doi:10.3389/fmicb.2021.657524)
Supplement: Supplementary file 1 [file Presentation_1.pdf]

Table S1. *Salmonella* colonies were recovered from soil models following treatment with phages Sergei, or Sergei and Munch in combination (see Fig. 4) and tested to determine their phage sensitivity. No phage-insensitive colonies were recovered from soil treated under either condition, at 1 hr or 24 hr following treatment.

Phage treatment	Treatment duration	No. colonies collected	No. colonies insensitive to treatment phage
**Sergei**	1 h	10	0
	24 h	20	0
**Sergei + Munch**	1 h	10	0
	24 h	20	0

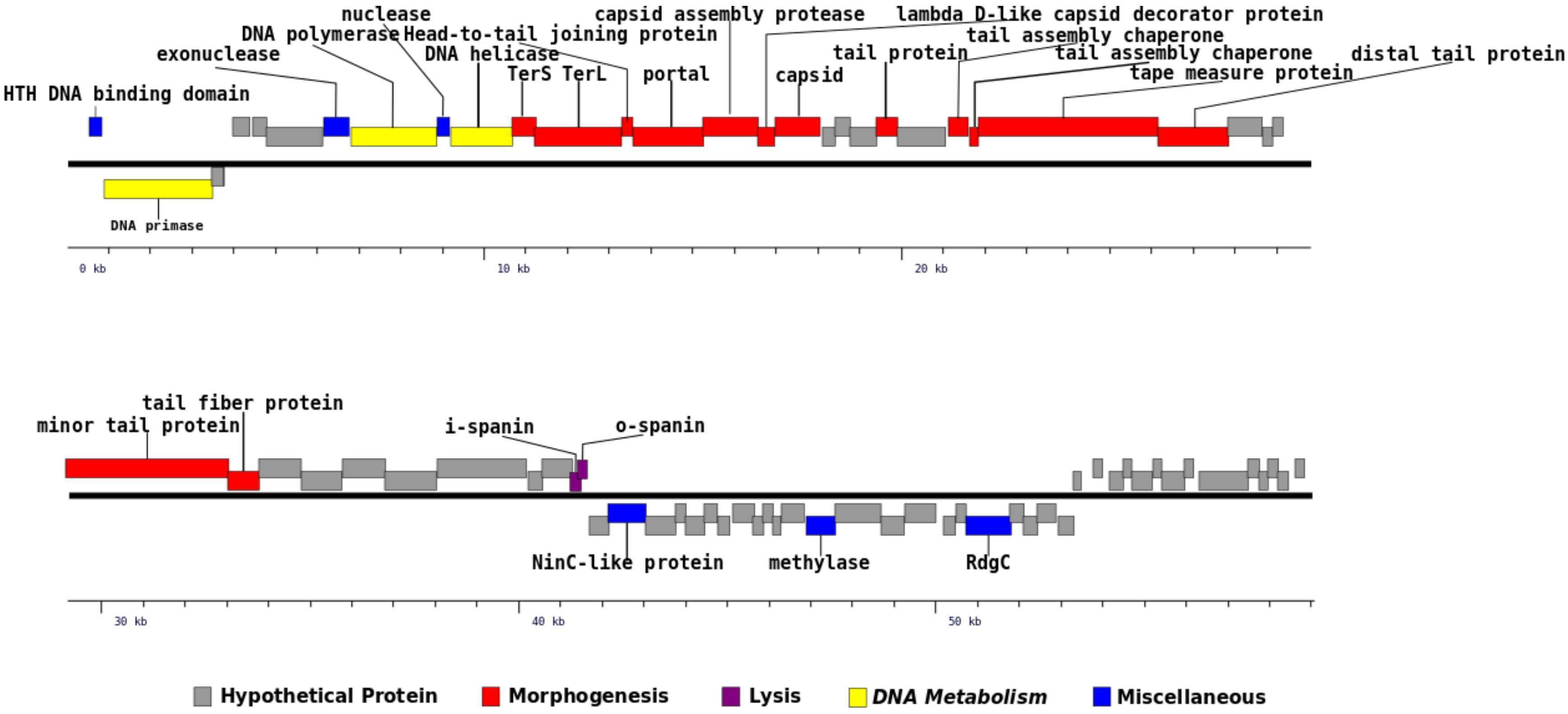

Figure S1. Genome map of phage Season12. Genes transcribed from the plus strand are drawn as boxes above the heavy black line, and genes transcribed from the minus strand are drawn below. Gene functions are labelled and color-coded by functional category as indicated in the legend.

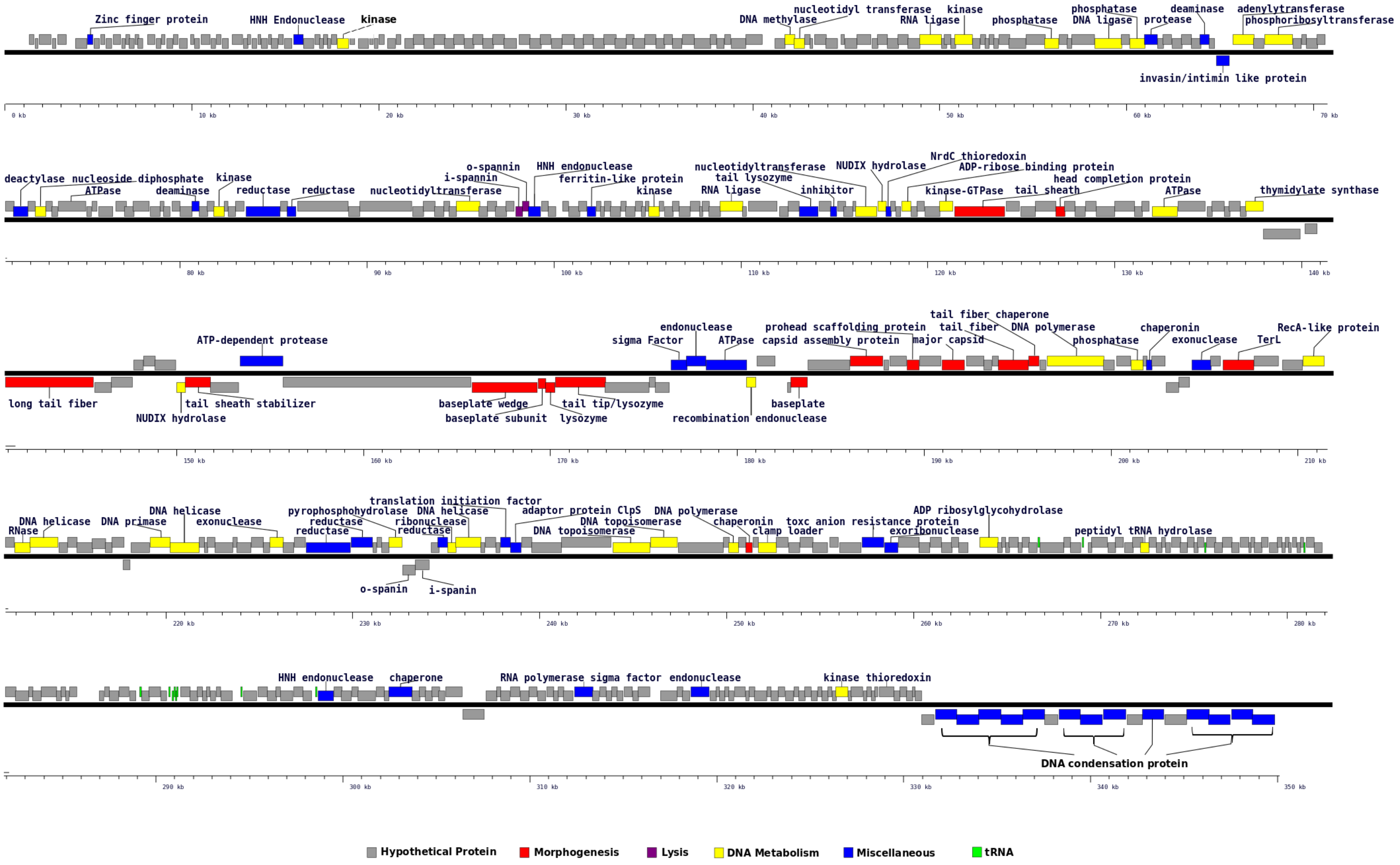

Figure S2. Genome map of jumbo phage Munch. Genes transcribed from the plus strand are drawn as boxes above the heavy black line, and genes transcribed from the minus strand are drawn below. Gene functions are labelled and color-coded by functional category as indicated in the legend.

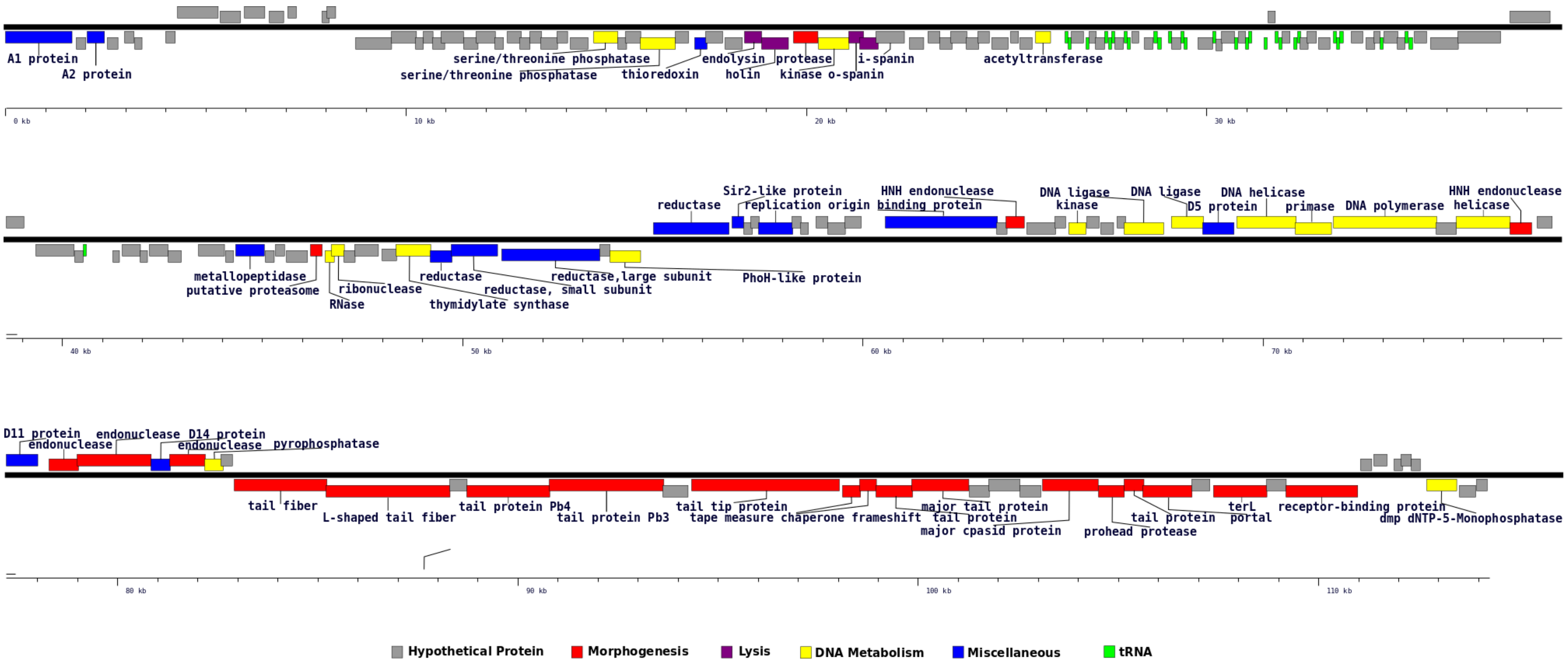

Figure S3. Genome map of phage Sw2. Genes transcribed from the plus strand are drawn as boxes above the heavy black line, and genes transcribed from the minus strand are drawn below. Gene functions are labelled and color-coded by functional category as indicated in the legend.

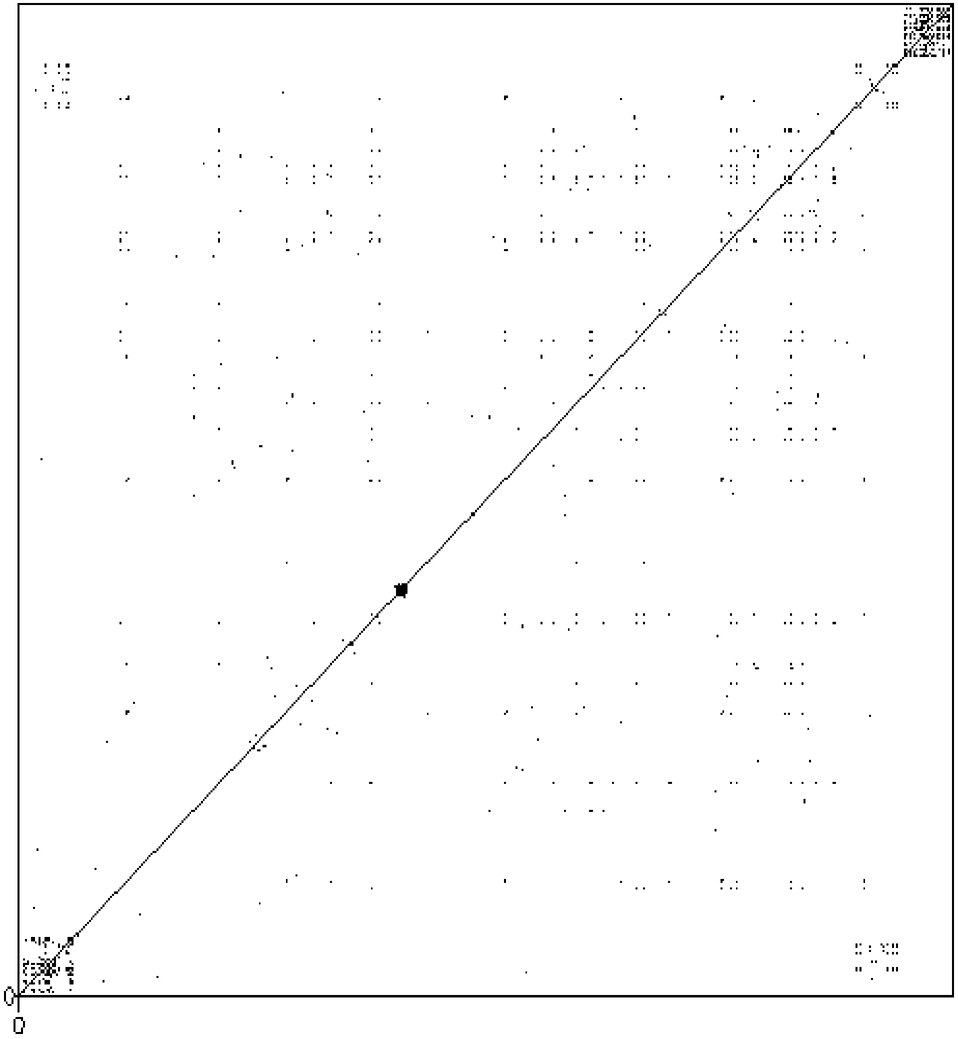

Figure S4. DNA dot plot obtained by mapping the sequence of jumbo phage Munch against itself with EMBOSS Dotmatcher. The dot plot shows regions with repetitive DNA sequence at the extreme left and right ~20 kb of the genome and within the predicted phage tail fiber gene (~141000-145000).
